# Supplementary material for: Uncovering protein glycosylation dynamics and heterogeneity using deep quantitative glycoprofiling (DQGlyco)
Source: Nat Struct Mol Biol. 2025 Feb 10;32(6):1111–26. doi: 10.1038/s41594-025-01485-w (PMC12170336; doi:10.1038/s41594-025-01485-w)
Supplement: Supplementary file 1 — Supplementary Figs. 1–9. [file 41594_2025_1485_MOESM1_ESM.pdf]

nature structural & molecular biology

Supplementary information https://doi.org/10.1038/s41594-025-01485-w

Uncovering protein glycosylation dynamics and heterogeneity using deep quantitative glycoprofiling (DQGlyco)

In the format provided by the authors and unedited

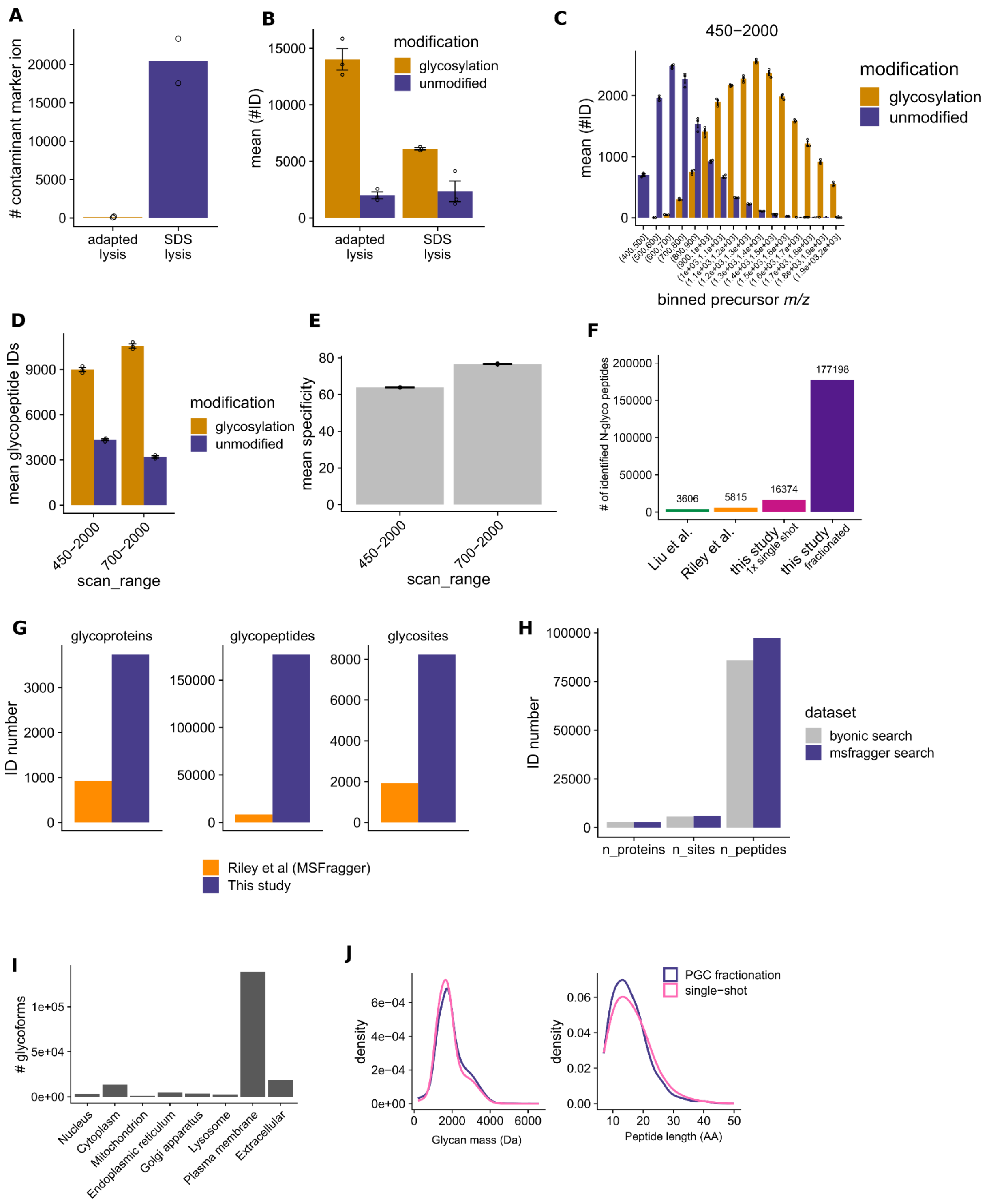

Supplementary Figure 1: DQGlyco optimization

A Mean number of contaminant ions (m/z 330.06) identified using a standard SDS lysis in comparison to an adapted optimized lysis in HEK293T cells. All samples were analyzed in duplicates. **B** Mean number of unique N-glycopeptides and unmodified peptides identified using a standard SDS lysis in comparison to an adapted optimized lysis in HEK293T cells. All samples were analyzed in triplicates. Data are presented as mean values +/- sd. **C** Mean number of N-glycopeptides and unmodified peptides identified in HEK293T per scan range

bin from 400-2000 m/z. All samples were analyzed in triplicates. Data are presented as mean values +/- sd. **D** Mean number of N-glycopeptides and unmodified peptides identified for measurements with two different scan ranges in HEK293T cells. All samples were analyzed in triplicates. Data are presented as mean values +/- sd. **E** Mean specificity (number of N-glycopeptide spectrum matches divided by all peptide spectrum matches) for measurements with two different scan ranges in HEK293T cells. Data are presented as mean values +/- sd. **F** Total number of unique N-glycopeptides (unique sequence and glycan composition) identified in this study and two recent N-glycoproteomic studies using off-line fractionation. **G** Number of unique N-glycoproteins, N-glycosites and N-glycopeptides identified in mouse brain samples in the Riley et al. dataset and in this study, when both datasets are processed with the same data analysis pipeline (with MS-Fragger as database search engine, with same glycan composition database). **H** Number of N-glycoproteins, N-glycosites and N-glycopeptides identified by MS-Fragger or Byonic search engines (same glycan database) for a PGC fractionation experiment of mouse brain sample. **I** Number of unique N-glycopeptides per protein subcellular annotation. **J** Distribution of N-glycan mass (Da) and N-glycopeptide amino acid length for single shot compared to PGC fractionated mouse brain samples

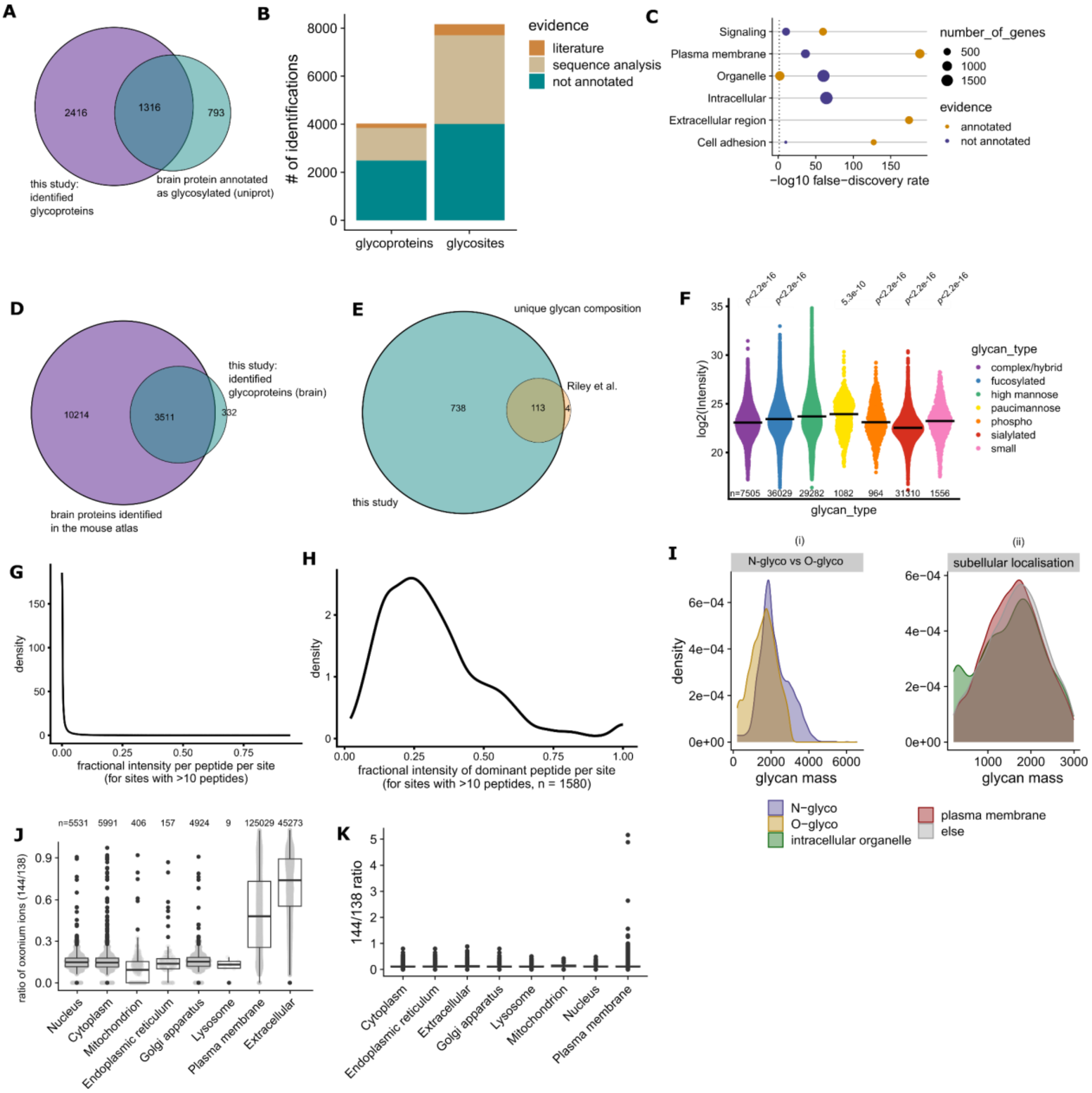

Supplementary Figure 2: Characterization of identified N- and O-glycoforms

A Overlap of glycoproteins identified in this study and proteins identified in the mouse brain which are annotated as glycosylated in the Uniprot database. **B** Uniprot annotation of glycoproteins identified in mouse brain samples. Literature evidence corresponds to experimental evidence while the majority of sites is annotated based on sequence analysis, i.e. prediction of glycosite based on the presence of the N-X-S/T, X≠P, glycosylation sequons. **C** Results of the Gene Ontology enrichment analysis (stringdb) of N-glycoproteins with and without annotation in the uniprot database. **D** Overlap of all proteins identified in the mouse brain of the mouse atlas and brain glycoproteins identified in this study. **E** Overlap of unique N-glycan compositions identified in this study and in the Riley et al.[1] study. **F** Log2 MS1 intensities of N-glycopeptides, grouped by glycan classes. Distributions were compared to the distribution of high mannose glycopeptide intensities (two-sided t-test). The horizontal lines

represent the median. **G** Density of fractional intensity of N-glycopeptides (MS1 intensity divided by the sum of MS1 intensities of all glycopeptides mapping to the same glycosite, for sites having more than 10 glycopeptides). **H** Density of fractional intensity of the most abundant N-glycopeptide per glycosite, for sites having more than 10 glycopeptides. **I** Frequency of (i) O- and N-glycopeptides per glycan mass, (ii) O-glycopeptides of proteins assigned to a specific Gene Ontology term per glycan mass. **J** 144/138 oxonium ions ratio for O-glycopeptides, per protein subcellular annotation. Lower ratio indicates the presence of GlcNac while higher ratio indicates the presence of GalNAc. Boxplots indicate median, first and third quartiles. Whiskers extend from the hinges to the largest value no further than 1.5× the interquartile range. Data points beyond the end of the whiskers are plotted individually. **K** 144/138 oxonium ions ratio for N-glycopeptides, per subcellular annotation.The horizontal lines represent the median.

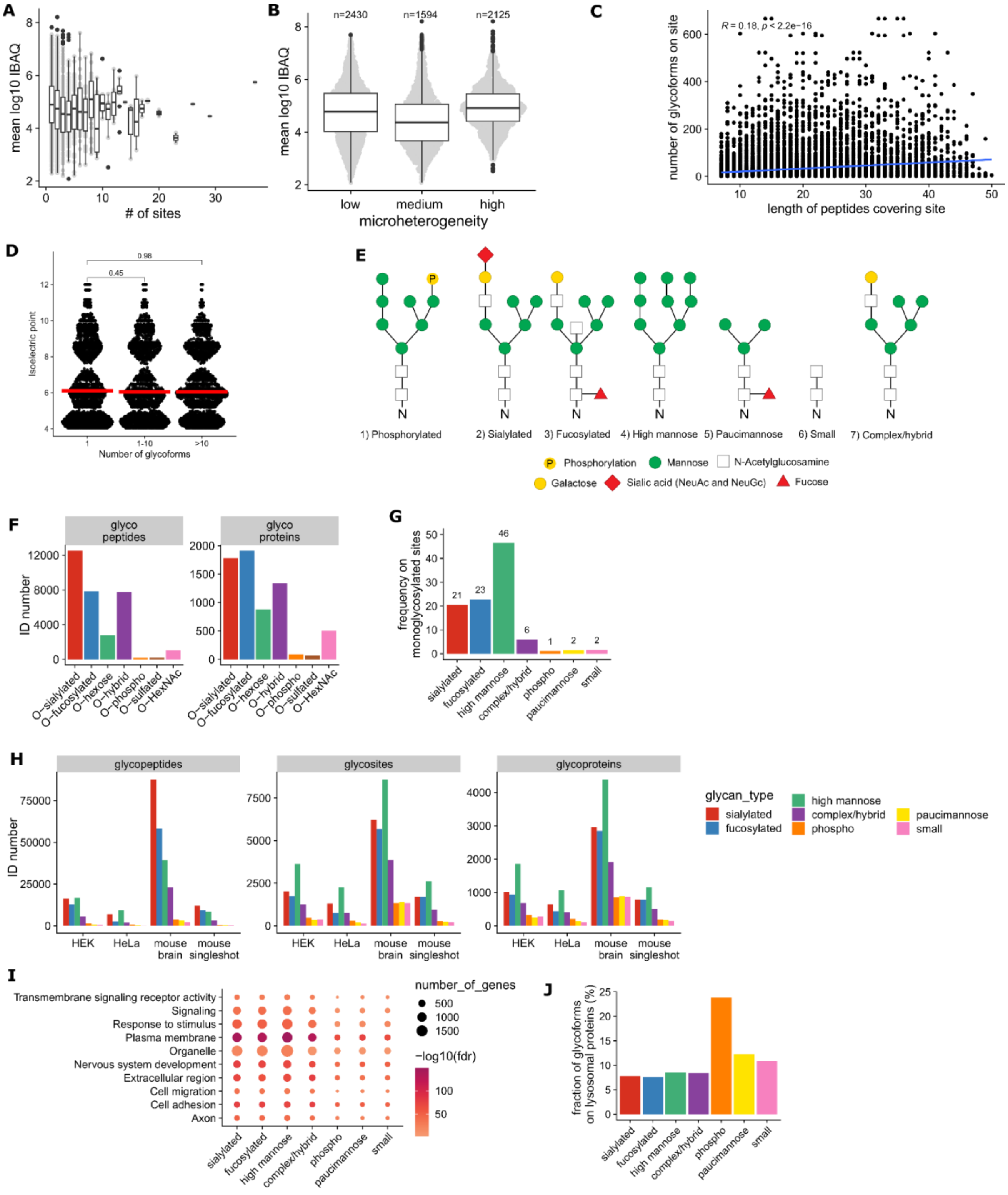

Supplementary Figure 3: Characterization of N- and O-glycan classes

A Estimated abundances (IBAQ values, extracted from the mouse proteome atlas) of glycoproteins quantified in the mouse brain compared with the number of sites per protein identified in this study. Boxplots indicate median, first and third quartiles. Whiskers extend from the hinges to the largest value no further than 1.5× the interquartile range. Data points beyond the end of the whiskers are plotted individually. **B** Microheterogeneity of sites compared to the mean estimated abundance of the corresponding protein quantified in the

mouse brain. Boxplots indicate median, first and third quartiles. Whiskers extend from the hinges to the largest value no further than 1.5× the interquartile range. Data points beyond the end of the whiskers are plotted individually. **C** Number of glycoforms detected per glycopeptide sequence as a function of peptide length (n = 12901 peptide sequences). The blue trend line corresponds to a linear model. **D** Isoelectric point of N-glycopeptides, grouped in 3 equally sized bins based on level of site microheterogeneity. N=4615, N=3660 and N=4626 for 1, 1-10 and >10 glycoforms, respectively. The horizontal lines represent the median. P values indicate the comparison of distributions via two-sided Wilcoxon Rank Sum test. **E** N-Glycan hierarchical classification used in this study. **E** Total number of unique O-glycopeptides and O-glycoproteins per glycan class for the PGC fractionated mouse brain sample. **F** Frequency of N-glycosites for which only one glycan composition was identified in the PGC fractionated mouse brain sample per glycan class. **G** Fraction of glycoforms belonging to each N-glycan class in the different subcellular compartments. **H** Total number of unique N-glycopeptides, N-glycosites and N-glycoproteins per glycan class for HEK293T cells, HeLaK cells, mouse brain single shot and the PGC fractionated mouse brain samples. **I** Results of the Gene Ontology enrichment analysis of N-glycoproteins with sites that are modified with different glycan classes (stringdb enrichment). **J** Frequency of N-glycopeptides that map to lysosomal proteins per glycan class.

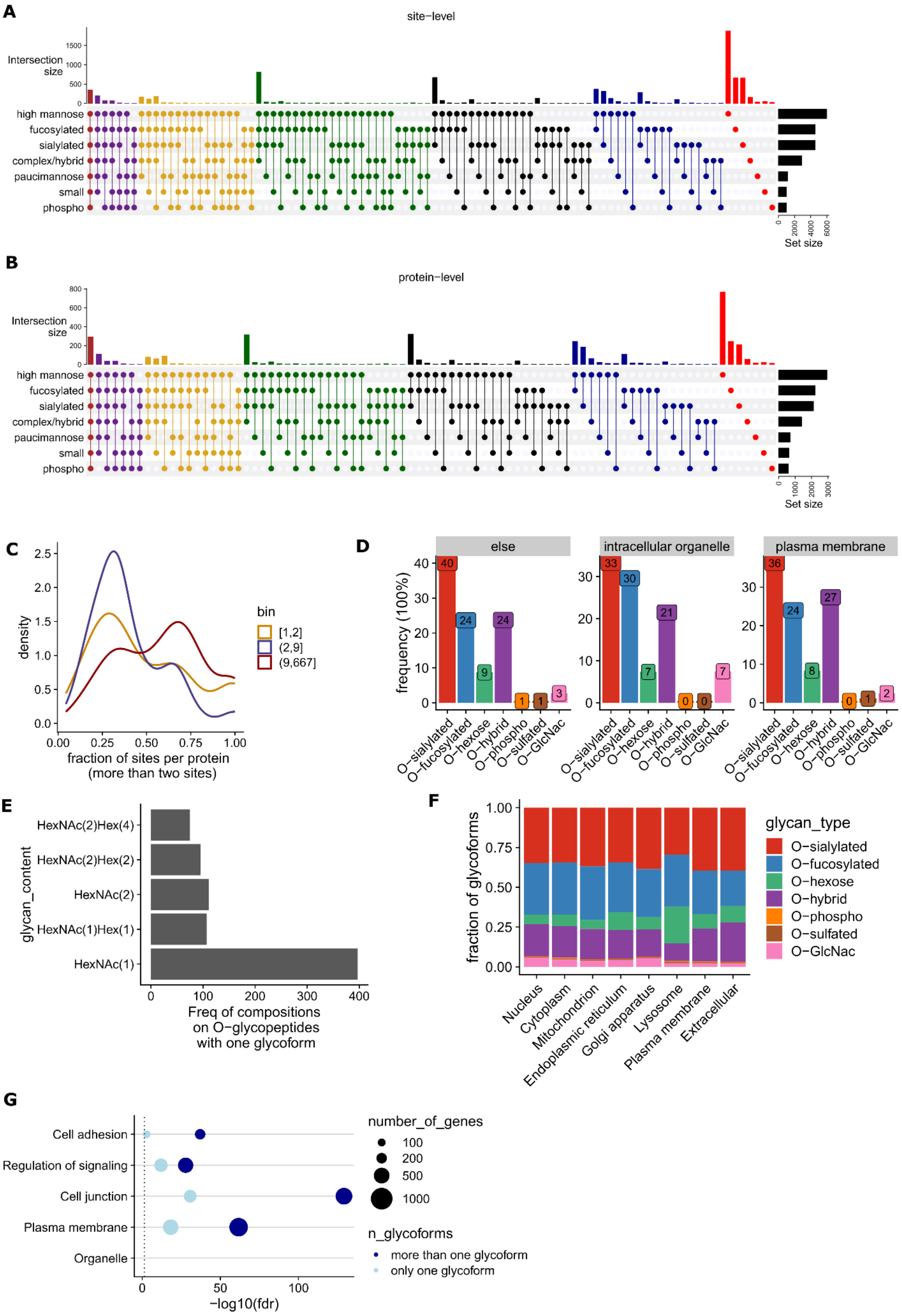
A
site−level
Intersection size
high mannose
fucosylated
sialylated
complex/hybrid
paucimannose
small
phospho
Set size
B
protein−level
Intersection size
high mannose
fucosylated
sialylated
complex/hybrid
paucimannose
small
phospho
Set size
C
density
bin
[1,2]
(2,9]
(9,667]
fraction of sites per protein
(more than two sites)
D
else
intracellular organelle
plasma membrane
frequency (100%)
O−sialylated
O−fucosylated
O−hexose
O−hybrid
O−phospho
O−sulfated
O−GlcNac
E
glycan_content
HexNAc(2)Hex(4)
HexNAc(2)Hex(2)
HexNAc(2)
HexNAc(1)Hex(1)
HexNAc(1)
Freq of compositions
on O−glycopeptides
with one glycoform
F
fraction of glycoforms
Nucleus
Cytoplasm
Mitochondrion
Endoplasmic reticulum
Golgi apparatus
Lysosome
Plasma membrane
Extracellular
glycan_type
O−sialylated
O−fucosylated
O−hexose
O−hybrid
O−phospho
O−sulfated
O−GlcNac
G
Cell adhesion
Regulation of signaling
Cell junction
Plasma membrane
Organelle
−log10(fdr)
number_of_genes
100
200
500
1000
n_glycoforms
more than one glycoform
only one glycoform

Supplementary Figure 4: Characterization of N- and O-glycosite microheterogeneity

A Co-occurrence of glycan classes on N-glycosites displayed as an Upset Plot. **B** Co-occurrence of glycan classes on N-glycoproteins displayed as an Upsetplot. **C** Fraction of sites within the same protein (here only proteins with more than 2 sites were considered) for the 3 microheterogeneity bins. **D** Frequencies of glycan classes of O-glycoproteins for selected Gene Ontology terms. **E** Frequencies of O-glycan compositions identified on O-glycopeptides on which only one glycoform was identified. **F** Fraction of glycoforms belonging to each O-glycan class in the different subcellular compartments. **G** Gene Ontology enrichment results (stringdb) for O-glycoproteins with sites that are either modified with only one glycan composition or with more than one glycan composition.

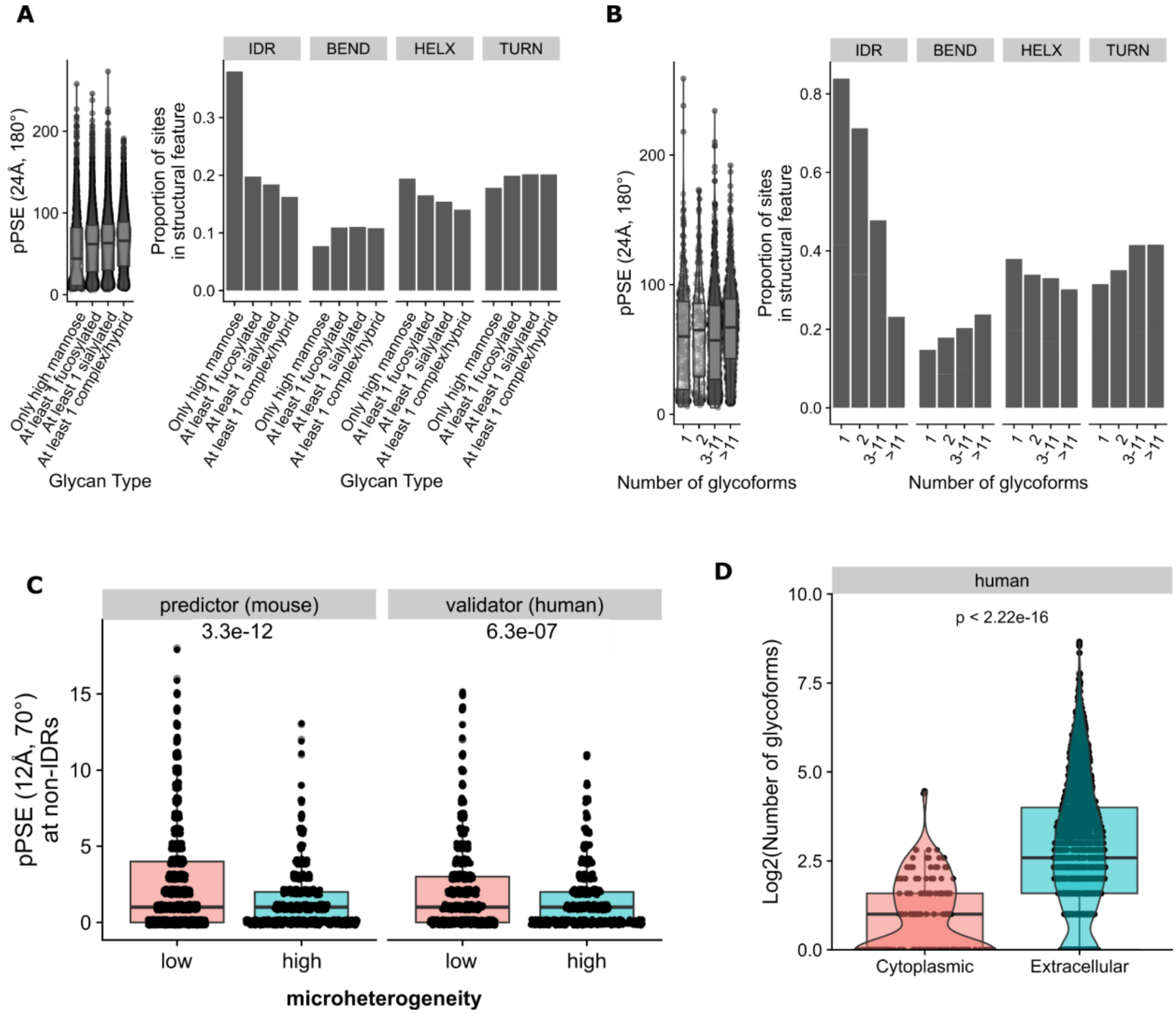

Supplementary Figure 5: Structural features used to predict N-glycosite microheterogeneity

Descriptive analysis of structural features of glycosylated asparagines identified by the deep glycoproteomics analysis of mouse brains. **A** Sites are grouped by glycan types: 1) Sites that contain only high mannose glycans (N=1873), 2) Sites that contain at least one fucosylated glycan (N=4560), 3) Sites containing at least one sialylated glycan (N=4550) and 4) Sites that contain at least one complex/hybrid glycan (N=2880). A site can be part of multiple categories if it contains multiple glycan types. Left side boxplots depict the prediction-aware part-sphere exposure (pPSE, 24Å, 180°) for sites on each category. Right side barplots indicate the proportion of sites that are part of a given structural feature. Vertical facets in the barplot indicate structural features: IDR = Intrinsically Disordered Region, BEND = Bend, HELX = Alpha-helix and TURN = Turn. **B** Same as A, but sites are grouped by the number of glycans identified on them (1; N=1642, 2; N=654, 3-11; N=2105, >11; N=1329). In this case, sites can not be part of multiple categories. **C** pPSE (12Å, 70°) distribution for lowly and highly glycosylated sites located at non-IDRs (mouse-low; N=1292, mouse-high; N=677, human-low; N=687, human-high; N=447). **D** Log transformed number of glycoforms on asparagines within

extracellular (N=1956) or cytoplasmic (N=164) topological domains for human cell line data. All boxplots indicate median, first and third quartiles. Whiskers extend from the hinges to the largest value no further than 1.5× the interquartile range. Data points beyond the end of the whiskers are plotted individually. All P values indicate the comparison of distributions via two-sided Wilcoxon Rank Sum test.

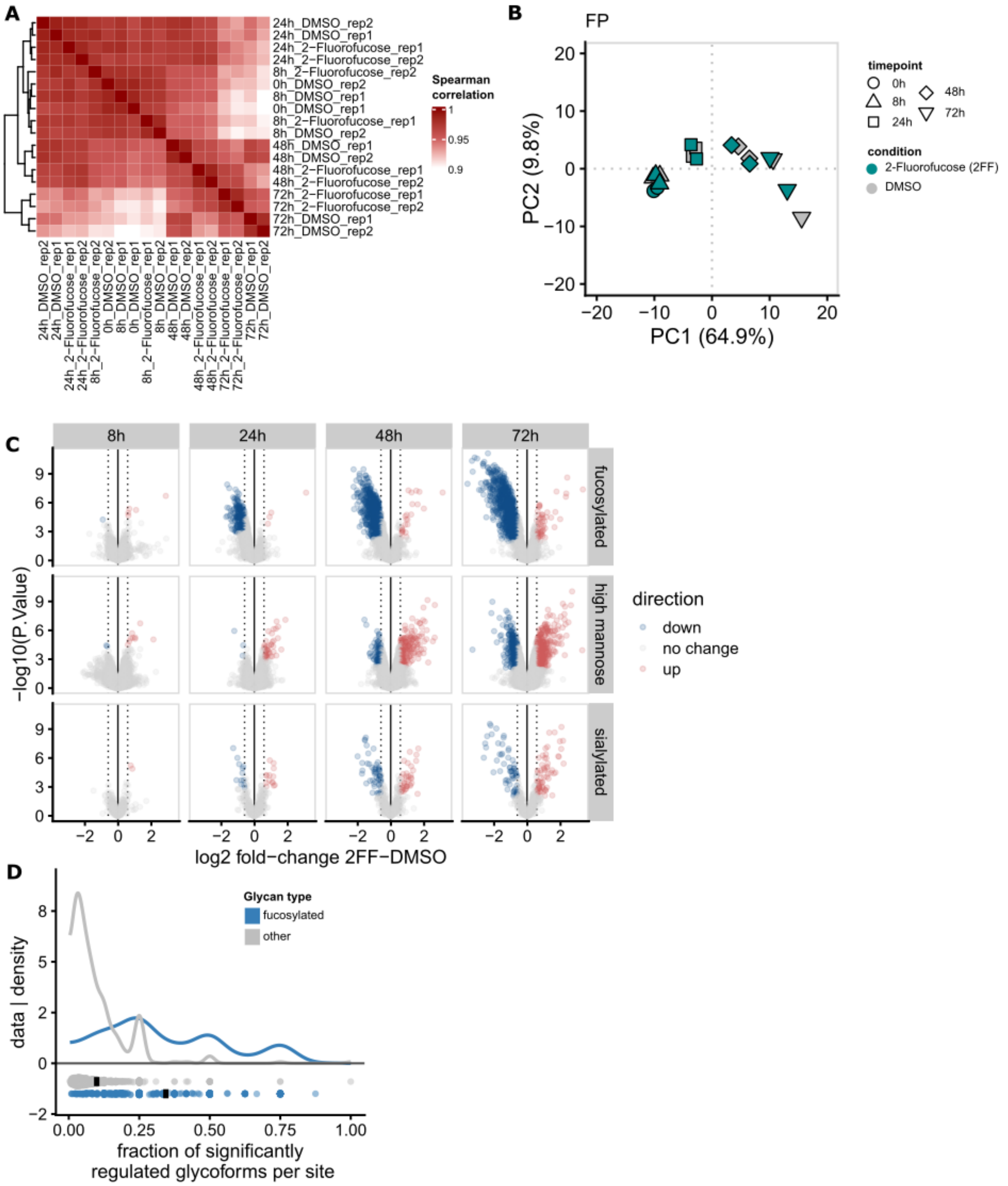

Supplementary Figure 6: Modulation of N-glycosylation upon fucosylation inhibition

A Spearman correlation of raw N-glycopeptides intensities between replicates. **B** Principal component analysis of the normalized TMT proteins intensities. **C** Regulation of glycopeptides upon treatment (2FF treated cells vs DMSO control cells at the different time points). The glycopeptides belonging to the fucosylated, high mannose and sialylated glycan classes are

displayed. **D** Fraction of significantly regulated glycoforms on a given site, for both fucosylated glycoforms and glycoforms belonging to the other glycan classes.

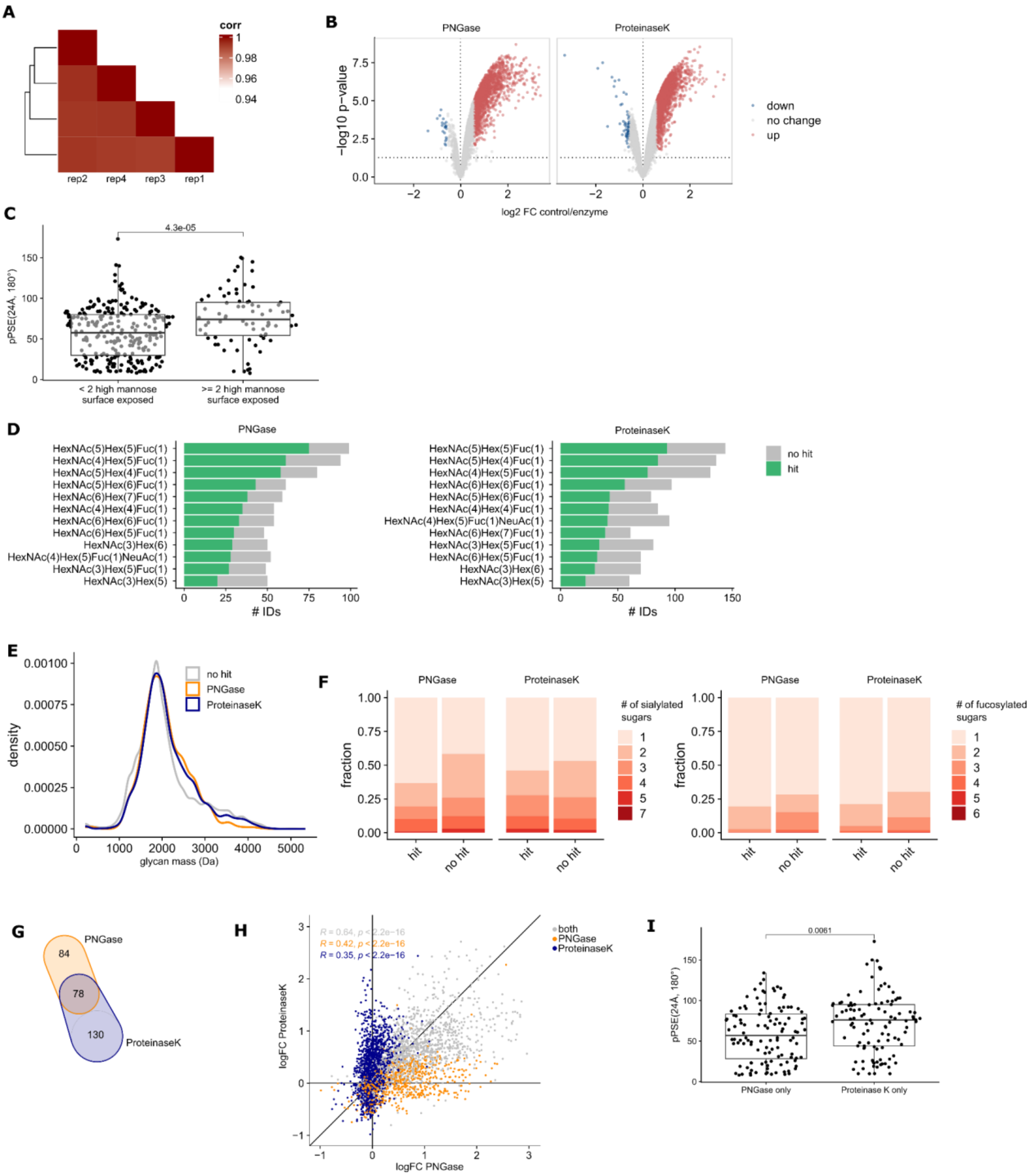

Supplementary Figure 7: Characterization of surface-exposed N-glycoforms

A Spearman correlation of glycopeptides abundance between 4 HEK293 cells replicates. **B** Comparison of glycopeptides abundance between intact living HEK293 cells treated with PNGase or Proteinase K and their respective controls. **C** Prediction-aware Part Sphere Exposure (pPSE) values of glycosites for which less than 2 (N=275) or at least 2 high mannose glycan compositions (N=85) were considered to be surface-exposed. **D** Glycan compositions most frequently identified as surface-exposed (hit) in HEK293 intact living cells treated with either PNGase or Proteinase K. **E** Distribution of glycan mass (Da) of glycopeptides identified as being intracellular (no hit) or surface-exposed in HEK293 cells treated with either PNGase

or Proteinase K. **F** Number of sialylated (left) and fucosylated (right) sugars in glycan compositions of glycopeptides in PNGase and Proteinase K experiments. **G** Overlap of glycosites exhibiting at least two glycopeptides changing in abundance upon PNGase or Proteinase K treatments. **H** Comparison of fold changes of glycopeptides abundance upon proteinase K (y-axis) and PNGase treatments (x-axis), for sites affected by either Proteinase K, PNGase or both enzymes (sites with at least two glycopeptides significantly changing in abundance upon treatment). **I** pPSE values of sites affected either only by PNGase (N=123) or only by Proteinase K (N=128) treatment. Boxplots indicate median, first and third quartiles. Whiskers extend from the hinges to the largest value no further than 1.5× the interquartile range. Data points beyond the end of the whiskers are plotted individually. All P values indicate the comparison of distributions via two-sided Wilcoxon Rank Sum test.

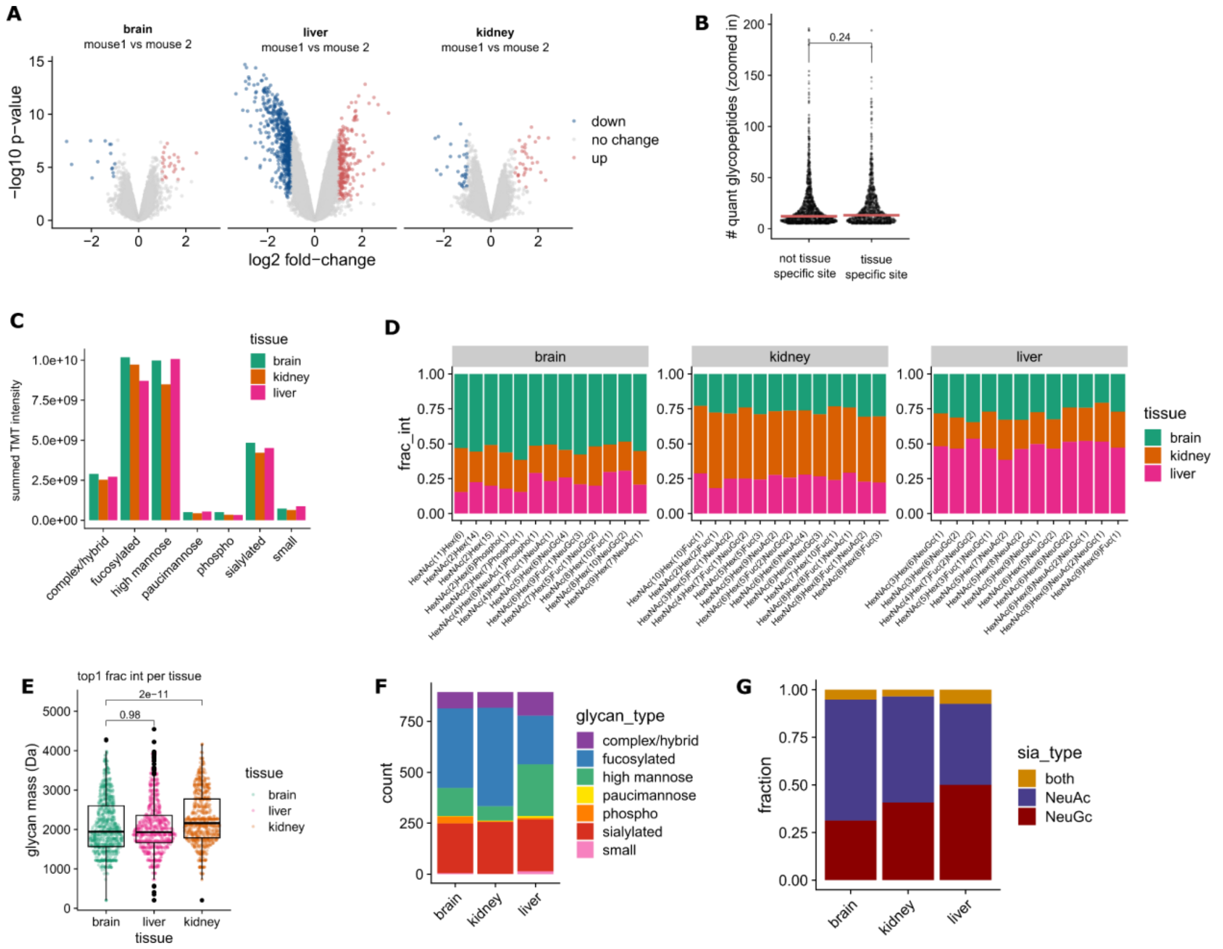

Supplementary Figure 8: N-glycosylation tissue specificity

A Abundance changes of glycopeptides in the same tissue across two mice. **B** Number of quantified glycopeptides for sites considered as tissue-specific (n = 1514) or non-tissue specific (n = 2012). The horizontal red lines correspond to the median. No difference was observed between the number of identified glycopeptides for tissue-specific and non-specific sites (two-sided Wilcoxon rank sum test, p-value > 0.05). **C** Summed TMT reporter ion intensities of all glycopeptides, per glycan class and per tissue. **D** Top 10 glycan compositions with the highest fractional intensity in each tissue. Only compositions identified at least 50 times were considered. **E** Distribution of glycan mass (Da) of the glycopeptides having the highest fractional intensity in each tissue (99th percentile, n = 894). The glycan mass of glycopeptides in kidney tissue is significantly higher compared to the brain and liver (two-sided Wilcoxon rank sum test, p-value < 0.05). Boxplots indicate median, first and third quartiles. Whiskers extend from the hinges to the largest value no further than 1.5× the interquartile range. Data points beyond the end of the whiskers are plotted individually. **F** Glycan classes of the glycopeptides having the highest fractional intensity in each tissue (99th percentile). **G** Type of sialylated sugar (NeuAc, NeuGc, or both) present in the compositions of sialylated glycopeptides having the highest fractional intensity in each tissue (99th percentile).

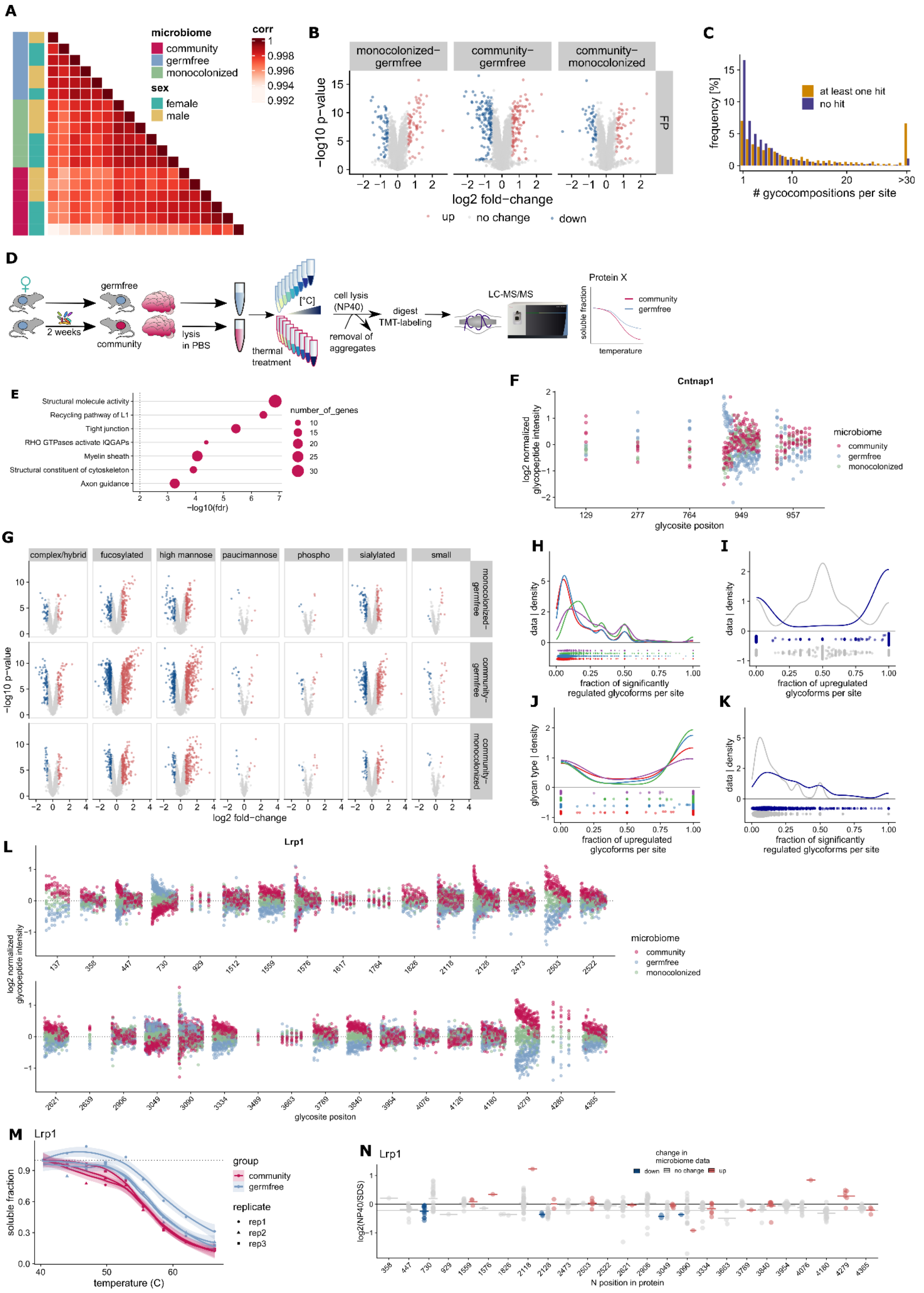
A
microbiome
community
germfree
monocolonized
sex
female
male
corr
1
0.998
0.996
0.994
0.992
B
monocolonized−germfree
community−germfree
community−monocolonized
FP
−log10 p−value
log2 fold−change
up
no change
down
C
frequency [%]
at least one hit
no hit
gycocompositions per site
D
germfree
2 weeks
community
lysis in PBS
[°C]
thermal treatment
cell lysis (NP40)
removal of aggregates
digest TMT-labeling
LC-MS/MS
Protein X
soluble fraction
temperature
E
Structural molecule activity
Recycling pathway of L1
Tight junction
RHO GTPases activate IQGAPs
Myelin sheath
Structural constituent of cytoskeleton
Axon guidance
−log10(fdr)
number_of_genes
F
Cntnap1
log2 normalized glycopeptide intensity
glycosite positon
microbiome
G
complex/hybrid
fucosylated
high mannose
paucimannose
phospho
sialylated
small
−log10 p-value
log2 fold−change
H
data | density
fraction of significantly regulated glycoforms per site
I
data | density
fraction of upregulated glycoforms per site
J
glycan type | density
fraction of upregulated glycoforms per site
K
data | density
fraction of significantly regulated glycoforms per site
L
Lrp1
log2 normalized glycopeptide intensity
glycosite positon
microbiome
community
germfree
monocolonized
M
Lrp1
soluble fraction
temperature (C)
group
community
germfree
replicate
rep1
rep2
rep3
N
Lrp1
change in microbiome data
down
no change
up
log2(NP40/SDS)
N position in protein

Supplementary Figure 9: Remodeling of the brain N-glycoproteome upon gut microbiome colonization

A Spearman correlation of protein intensities between biological replicates. **B** Protein-level regulation in the mouse brain depending on the gut microbiome composition. **C** Number of unique glycan compositions per site for sites exhibiting at least one significantly regulated glycoform or no glycoform regulated. **D** Thermal Proteome Profiling (TPP) workflow. **E** GO terms enrichment for proteins exhibiting significant change in thermal stability upon gut microbiome colonization (stringdb enrichment, FDR < 0.05). **F** Glycopeptides on protein Cntnap1 significantly change in abundance upon gut-microbiome colonization. **G** Regulation of glycopeptides, with glycopeptides being grouped by glycan class. **H** Fraction of glycoforms which are regulated significantly per site with at least two glycoforms (blue) compared to randomly assigned regulation annotation (grey). On sites with multiple glycoforms detected only a small fraction of glycoforms changes significantly depending on the gut microbiome composition, suggesting that there is no general regulation at the glycosite level. **I** The fraction of glycoforms which are regulated significantly per site and glycan class with at least two glycoforms per class indicates that glycosite level regulation does not depend on a certain glycan class (i.e all fucosylated forms on one site are significantly upregulated). Only glycan types with enough data points shown. **J** Fraction of glycoforms which are upregulated out of all significantly regulated glycoforms per site compared to randomly assigned regulation direction (grey). For glycosites with at least one significantly regulated glycoform, most regulated glycoforms tended in the same direction of regulation, as opposed to random direction of regulation. **K** This trend for the direction of regulation can also be observed for different glycan classes. Only glycan types with enough data points shown. **L** Example of site-specific regulation of N-glycosylation on the prolow-density lipoprotein receptor-related protein 1 (Lrp1). Each vertical line represents one glycopeptide while each dot represents a replicate (3 conditions x 6 biological replicates). **M** Lrp1 protein demonstrates a change in thermal stability upon gut-microbiome colonization with the 8-member community. Data are presented as mean values +/- sd. **N** Solubility of the Lrp1 glycoforms measured in the SPP experiment. Glycoforms modulated upon gut-microbiome colonization are highlighted.The horizontal lines represent the median.

Supplementary References

1. Riley, N. M., Hebert, A. S., Westphall, M. S. & Coon, J. J. Capturing site-specific heterogeneity with large-scale N-glycoproteome analysis. *Nat. Commun.* **10**, 1311 (2019).
