# Supplementary material for: Uncovering protein glycosylation dynamics and heterogeneity using deep quantitative glycoprofiling (DQGlyco)
Source: Nat Struct Mol Biol. 2025 Feb 10;32(6):1111–26. doi: 10.1038/s41594-025-01485-w (PMC12170336; doi:10.1038/s41594-025-01485-w)
Supplement: Supplementary file 2 — Reporting Summary [file 41594_2025_1485_MOESM2_ESM.pdf]

nature portfolio

Corresponding author(s): Mikhail Savitski
Clement Potel

Last updated by author(s): Dec 16, 2024

Reporting Summary

Nature Portfolio wishes to improve the reproducibility of the work that we publish. This form provides structure for consistency and transparency in reporting. For further information on Nature Portfolio policies, see our Editorial Policies and the Editorial Policy Checklist.

Statistics

For all statistical analyses, confirm that the following items are present in the figure legend, table legend, main text, or Methods section.

n/a	Confirmed	
☐	☒	The exact sample size (*n*) for each experimental group/condition, given as a discrete number and unit of measurement
☐	☒	A statement on whether measurements were taken from distinct samples or whether the same sample was measured repeatedly
☐	☒	The statistical test(s) used AND whether they are one- or two-sided<br>*Only common tests should be described solely by name; describe more complex techniques in the Methods section.*
☐	☒	A description of all covariates tested
☐	☒	A description of any assumptions or corrections, such as tests of normality and adjustment for multiple comparisons
☐	☒	A full description of the statistical parameters including central tendency (e.g. means) or other basic estimates (e.g. regression coefficient) AND variation (e.g. standard deviation) or associated estimates of uncertainty (e.g. confidence intervals)
☐	☒	For null hypothesis testing, the test statistic (e.g. *F*, *t*, *r*) with confidence intervals, effect sizes, degrees of freedom and *P* value noted<br>*Give P values as exact values whenever suitable.*
☒	☐	For Bayesian analysis, information on the choice of priors and Markov chain Monte Carlo settings
☒	☐	For hierarchical and complex designs, identification of the appropriate level for tests and full reporting of outcomes
☐	☒	Estimates of effect sizes (e.g. Cohen's *d*, Pearson's *r*), indicating how they were calculated

Our web collection on statistics for biologists contains articles on many of the points above.

Software and code

Policy information about availability of computer code

Data collection	Vendor acquisition softwares: Thermo Scientific Xcalibur 4.6 and Tune 4.2
Data analysis	MSFragger v20<br>MSConvert 3.0.19057-5e3190638<br>Python 3.9.12<br>R 4.2.1<br>Structuremap package (v0.0.9)<br>XGBoost python package (v2.0.3)<br>All code used to perform the computational analyses described and to reproduce the figures is available at https://github.com/miralea/DQGlyco

For manuscripts utilizing custom algorithms or software that are central to the research but not yet described in published literature, software must be made available to editors and reviewers. We strongly encourage code deposition in a community repository (e.g. GitHub). See the Nature Portfolio guidelines for submitting code & software for further information.

Data

Policy information about availability of data

All manuscripts must include a data availability statement. This statement should provide the following information, where applicable:

- Accession codes, unique identifiers, or web links for publicly available datasets
- A description of any restrictions on data availability
- For clinical datasets or third party data, please ensure that the statement adheres to our policy

All raw files, databases (downloaded from Uniprot: Swissprot Homo sapiens database (20,443 entries) and Mus musculus reference proteome with one protein per gene sequence (22,084 entries)) and search outputs were deposited to the ProteomeXchange Consortium via the PRIDE partner repository with the dataset identifier PXD052447.

Human research participants

Policy information about studies involving human research participants and Sex and Gender in Research.

Reporting on sex and gender	NA
Population characteristics	NA
Recruitment	NA
Ethics oversight	NA

Note that full information on the approval of the study protocol must also be provided in the manuscript.

Field-specific reporting

Please select the one below that is the best fit for your research. If you are not sure, read the appropriate sections before making your selection.

☒ Life sciences ☐ Behavioural & social sciences ☐ Ecological, evolutionary & environmental sciences

For a reference copy of the document with all sections, see nature.com/documents/nr-reporting-summary-flat.pdf

Life sciences study design

All studies must disclose on these points even when the disclosure is negative.

Sample size	No sample size calculation was performed. All experiments were performed in triplicates to perform statistical analysis. No statistical methods were used to pre-determine sample sizes but our sample sizes are similar to those routinely reported in proteomics publications.
Data exclusions	Peptides and proteins with incomplete TMT quantification were excluded, as well as proteins that matched the contaminant database.
Replication	All proteomics experiments were conducted in biological triplicates and replications were successful.
Randomization	No randomization was performed, as standard for this type of experiments. Experiments were measured sequentially on mass spectrometers as is the general practice in proteomics.
Blinding	No blinding was performed as this study would not be impacted by blinding.

Reporting for specific materials, systems and methods

We require information from authors about some types of materials, experimental systems and methods used in many studies. Here, indicate whether each material, system or method listed is relevant to your study. If you are not sure if a list item applies to your research, read the appropriate section before selecting a response.

Materials & experimental systems

n/a	Involved in the study
☒	☐ Antibodies
☐	☒ Eukaryotic cell lines
☒	☐ Palaeontology and archaeology
☐	☒ Animals and other organisms
☒	☐ Clinical data
☒	☐ Dual use research of concern

Methods

n/a	Involved in the study
☒	☐ ChIP-seq
☒	☐ Flow cytometry
☒	☐ MRI-based neuroimaging

Eukaryotic cell lines

Policy information about cell lines and Sex and Gender in Research

Cell line source(s)	Hela-Kyoto (Schmitz et al., 2010) - was a gift from Jan Ellenberg's group, EMBL - original cell line (RRID: CVCL_1922) was received as gift from Prof. S. Narumiya, Kyoto University and authenticated by sequencing.<br>HEK293T cells (ATCC-CRL-3216) were obtained from the American Type Culture Collection (ATCC). Expi293F were obtained from Thermo Fisher Scientific. HEK293 and HeLa-Kyoto are not on the list of commonly misidentified cell lines.
Authentication	Hela-Kyoto cells have been authenticated by sequencing. HEK293 cells were not authenticated.
Mycoplasma contamination	All cell lines tested negative for mycoplasma contamination.
Commonly misidentified lines (See ICLAC register)	No commonly misidentified lines were used in this study.

Animals and other research organisms

Policy information about studies involving animals; ARRIVE guidelines recommended for reporting animal research, and Sex and Gender in Research

Laboratory animals	Germfree (GF) C57BL/6 mice were maintained and bred in gnotobiotic isolators (CbC) with a 12-hour light/dark cycle. GF status was monitored by PCR (using 16S primer pair) and culture-based methods. Mice were provided with standard, autoclaved chow (1318 P FORTI, Altromin) ad libitum. Animals of mixed gender at an age between 8 and 17 weeks were used for all experiments.
Wild animals	The study did not involved wild animals.
Reporting on sex	Mice of both male and female sex were used for this study.
Field-collected samples	The study did not involved samples collected from the field.
Ethics oversight	All mouse experiments were performed using approved protocols by the EMBL ethics committee (licence 21-002_HD_MZ).

Note that full information on the approval of the study protocol must also be provided in the manuscript.
